# Neural Dysconnectivity in the Hippocampus Correlates With White Matter Lesions and Cognitive Measures in Patients With Coronary Artery Disease

**DOI:** 10.3389/fnagi.2022.786253

**Published:** 2022-06-27

**Authors:** Jianhua Niu, Jingchen Zhang, Jueyue Yan, Zhipeng Xu, Xing Fang, Jingyu You, Zhihai Liu, Weifang Wu, Tong Li

**Affiliations:** Department of Critical Care Medicine, The First Affiliated Hospital, Zhejiang University School of Medicine, Hangzhou, China

**Keywords:** Montreal Cognitive Assessment (MoCA), Mini Mental State Examination (MMSE), Fazekas score, functional magnetic resonance imaging (fMRI), coronary artery disease (CAD)

## Abstract

**Purpose:**

Recent neuroimaging reports have shown the microstructural changes in coronary artery disease (CAD) and its correlation with cognitive dysfunction while little is known about the functional characteristics of CAD. We hypothesize that functional characteristics may give clues to underlying pathology in CAD and its link with cognitive dysfunction. Degree centrality (DC), a graph-based assessment of network organization was performed to explore the neural connectivity changes in CAD patients compared with healthy controls and their correlation with cognitive measures.

**Methods:**

Thirty CAD patients and 36 healthy controls were included in our study. All participants underwent functional magnetic resonance imaging (fMRI) of the brain. We performed DC analysis to identify voxels that showed changes in whole-brain functional connectivity with other voxels. DC was measured by the fMRI graph method and comparisons between the two groups were done. All participants underwent neuropsychological assessment (Montreal Cognitive Assessment, MoCA and Mini-Mental State Examination, MMSE).

**Results:**

Our data analysis included 30 CAD patients (59.90 ± 7.53 years) and 36 HCs (61.61 ± 6.19 years). CAD patients showed a greater prevalence of white matter lesions using the Fazekas score than healthy controls (*P* < 0.001). Importantly, CAD patients showed significantly lower (*P* < 0.001) MoCA and MMSE scores compared with healthy controls. CAD patients showed significantly decreased DC value (*P* < 0.001) in the right hippocampus (hippocampus_R), right lingual gyrus (lingual_R), and significantly increased DC value (*P* < 0.001) in the left middle frontal gyrus (Frontal_Mid_L) when compared with healthy controls respectively. DC value in the hippocampus_R significantly correlated (*P* < 0.00) with MMSE and MoCA scores in CAD patients. Fazekas scores in CAD patients showed a significant correlation (*P* < 0.001) with the DC value in the hippocampus_R.

**Conclusion:**

These findings suggest that reduced cerebral neural connectivity in CAD may contribute to their cognitive impairment and white matter microstructural damage.

## Introduction

Coronary artery disease (CAD) is caused by the build of plaque in the wall of arteries that supply blood to the heart; plaques cause narrowing or blockage that could reduce blood flow to the heart which may lead to a heart attack. Increasing evidence suggests an association between CAD and cognitive impairment even in the absence of ischemic stroke (Burkauskas et al., 2016, 2018; Lowenstern and Wang, 2019). The potential mechanism underlying this association include cerebral atherosclerosis and hypoperfusion, which may have been linked with cerebral small vessel disease (CSVD) (Berry et al., 2019).

Neuroimaging reports have shown that CAD is associated with white matter lesions (Ikram et al., 2008; Vidal et al., 2010), and cerebral infarcts (Geerlings et al., 2010) which are radiological indicators of small vessel disease; reports have also shown that CAD is associated with transient ischemic attacks (TIA) (Adams et al., 2003) and gray matter microstructural changes (DeCarli et al., 1999). Nonetheless, very little is known about the neural network changes that occur in the brain of CAD patients; understanding the underlying mechanisms may bring insight into the cerebral changes and cognitive impairment that occur during the disease mechanism.

Magnetic resonance imaging (MRI) reports have mostly focused on the microstructural changes that occur in CAD patients. CAD patients tend to present with reduced gray matter and white matter microstructure compared with controls (Almeida et al., 2012; Vuorinen et al., 2014; Anazodo et al., 2015); it is also suggested that CAD patients showed cortical thickness in multiple regions of the brain (Almeida et al., 2012; Vuorinen et al., 2014). Importantly, reports (Deckers et al., 2017; Elman et al., 2019; Faulkner et al., 2021; Liang et al., 2021) have shown the association between CAD and the risk for cognitive impairment or dementia but very little is known about the association between this link. However, it is suggested that the association between cognitive impairment and CAD may be linked with underlying risk factors (such as hypertension, diabetes mellitus which have been suggested to be linked with cognitive dysfunction), atherosclerosis, and hypoperfusion.

To date, very little is known about the functional characteristics of the brain in CAD patients which may give clues to the underlying mechanisms. Degree centrality (DC), assessed by functional magnetic resonance imaging (fMRI) has acquired incredible consideration lately. This graph-based assessment of network organization reflects the number of instantaneous functional connections between a region and the rest of the brain within the entire connectivity matrix of the brain. In that, DC can assess how much a node influences the entire brain and integrates information across functionally segregated brain regions. Voxel-wise centrality maps have provided novel insights into patterns and complexity of functional connectivity in Alzheimer’s disease (AD) (Guo et al., 2017).

Our current study focused on network architecture to investigate the intrinsic dysconnectivity pattern in whole-brain functional networks at the voxel level in CAD patients compared with healthy controls. We chose DC because its measures take into account a given region’s relationship with the entire functional connectome and not just its relation to individual regions or to separate larger components; therein, DC allows one to capture the complexity of the functional connectome as a whole. We also assessed the correlation between DC changes and their clinical cognitive assessment scores. We hypothesize that functional characteristics may give clues to underlying pathology in CAD and its link with cognitive dysfunction.

## Materials and Methods

This observational cross-sectional study was done at the First Affiliated Hospital of Zhejiang University School of Medicine from September 2020 to July 2021. The inclusion criteria for CAD patients were as follows: (1) age between 35 and 80 years; (2) diagnosed with CAD (Johansen et al., 2021); and (3) could cooperate during magnetic resonance imaging. The exclusion criteria were as follows: the presence of carotid artery stenosis or pseudo-occlusion, stroke, and patients who could not cooperate during MR imaging.

The control group was individuals who attended our hospital for annual health check-ups and had no history of neurologic or cardiovascular diseases.

All participants were evaluated for cardiovascular risk factors, medical history, medication use and had a comprehensive cardiovascular physical examination by a cardiologist. The study was approved by the Ethics Committee of First Affiliated Hospital of Zhejiang University School of Medicine. Participants recruited provided written informed consent before enrolling in the study.

### Neuropsychological Examinations

All participants underwent a Montreal Cognitive Assessment (MoCA) and Mini-Mental State Examination (MMSE) which are examinations to screen for cognitive decline. These examinations have a total score of 30 and a score lower than 26 indicates worse cognition in MoCA while a score lower than 24 indicates worse cognition in MMSE.

#### Magnetic Resonance Imaging Protocol

Whole-brain MRI data were acquired at the Center for Brain Imaging Science and Technology, First Affiliated Hospital of Zhejiang University School on a Siemens MAGNETOM Prisma 3T scanner (Siemens, Erlangen, Germany). All participants were placed in the machine with foam padding around the head to reduce motion; they were asked to keep still with their eyes closed during imaging.

An echo-planar imaging sequence was used to acquire the functional images with the following: 60 axial slices, thickness/gap = 2.0/0mm, in-plane resolution = 64 × 64, repetition time (TR) = 2,000 ms, echo time (TE) = 34 ms, flip angle = 62° and field of view (FOV) = 220 mm × 220 mm. Anatomical T1-weighted whole brain magnetization-prepared rapid gradient echo images were obtained using the following: 160 sagittal slices, slice thickness/gap = 1.2/0 mm, in-plane resolution = 512 × 512, TR = 5,000 ms, TE = 2.9 ms, inversion time (TI) = 700 ms, flip angle = 4° and FOV = 256 mm × 256 mm.

#### Processing of MRI Data

SPM8^1^ was used to implement pre-processing of all fMRI data while data processing was done with Data Processing Assistant for Resting-State fMRI.^2^ The initial 10 volumes of the functional images were discarded to remove initial transient effects and to allow the participant to adjust to the scanner noise before pre-processing. The rest of the fMRI images were acquired with slice timing for the acquisition delay between slices and correction of head motion. All participants who were under imaging had less than 1.5 mm maximum displacement in x, y, or z and 1.5° angular motion during imaging. Spatial normalization and resampling to 3 mm voxels were used to acquire realigned images while a Gaussian filter (6 mm FWHM) was used to spatially smoothen the images. Smoothened images were filtered using a typical temporal bandpass (0.01–0.08 Hz) to reduce low-frequency drift, physiological high-frequency respiratory and cardiac noise. Linear trends were removed within each time series. Lastly, spurious variances from several sources were removed by linear regression including six head motion parameters, along with average signals from cerebrospinal fluid and white matter.

#### Calculation of DC

Voxel-based whole-brain correlation analysis on pre-processed fMRI was done to calculate voxel-wise DC as previously described (Li et al., 2016). Pearson’s correlation coefficients (r) were done between all pairs of brain voxels in the gray matter mask. We then converted the Pearson’s correlation data to normally distributed Fisher’s Z-scores and constructed the whole-brain functional network by thresholding each correlation at r > 0.25 as previously reported (Buckner et al., 2009). DC for a given voxel was calculated as the sum of the significant connections at the individual level. Voxel-wise DC values were also converted into a Z-score map using the Fisher-Z transformation to improve normality. Positive correlations were considered in the DC calculation due to the uncertainty of interpretation and detrimental effects on test-retest reliability.

To assess the DC difference between CAD patients and HC, a two-sample t-test was performed using REST. AlphaSim, a program based on Monte Carlo simulation and implemented in AFNI,^3^ was used for multiple comparison corrections. Monte Carlo simulations determine the random distribution of cluster size for a given per voxel threshold (Ledberg et al., 1998). Statistical difference was defined as *P* < 0.05 and cluster size > 198 voxels, corresponding to a corrected *P* < 0.05. The correction was confined within the gray matter mask and was determined by Monte Carlo simulations (Ledberg et al., 1998).

### White Matter Lesion Rating Using the Fazekas Scale

White matter lesions (WML) were rated based on FLAIR and T2-W cerebral images using the Fazekas scale. Scores ranged from 0 to 3 as previously reported (Han et al., 2018). A modification of suggested rating scales was used to describe different types of hyperintense signal abnormalities around the ventricles (periventricular white matter hyperintensities, PWMH) and in the deep white matter (DWMH) as previously reported (Fazekas et al., 1987).

### Statistical Analysis

SPSS (version 24) was used for our statistical analysis. Continuous variables were displayed as mean ± standard deviation, number (%) as appropriate. To assess the correlation between DC changes and clinical features of CAD, multivariable linear regression was used while adjusting for risk factors (age, gender, hypertension, and dyslipidemia). *P* values less than 0.05 were considered statistically significant.

## Results

We initially enrolled 70 participants (34 CAD and 36 HCs), but 2 CAD patients were excluded because of uncooperating during MR imaging and 2 CAD patients were excluded because of cerebral infarction after MR imaging as shown in Supplementary Figure 1. Our data analysis included 30 CAD patients (59.90 ± 7.53 years) and 36 HCs (61.61 ± 6.19 years). Of the 30 CAD patients, 23 (76.67%) were males, 22 (73.33%) had a history of hypertension while 20 (66.67%) had a history of dyslipidemia. Table 1 shows the demographics and clinical information of our participants. CAD patients showed a greater prevalence of white matter lesions using the Fazekas score than healthy controls (*P* < 0.001, Table 1). Importantly, CAD patients showed significantly lower (*P* < 0.001, Table 1) MoCA and MMSE scores compared with healthy controls, respectively.

**TABLE 1:** Demographics and clinical data.

Variable	CAD (n = 30)	HC (n = 36)	*P*-value
**Demographic information**			
Male sex, No. (%)	23 (67.74%)	20 (80.5%)	0.672
Age (years), mean (SD)	59.90 ± 7.53	61.61 ± 6.19	0.865
Hypertension, No.	22	16	0.722
Diabetes mellitus, No.	0	0	0.909
Hypercholesterolemia, No.	20	15	0.448
Dyslipidemia, No.	20	16	0.468
LVEF,%	57.53 ± 4.16	59.69 ± 3.88	0.052
Smokers, No.	15	8	0.380
Education, years	5.07 ± 1.31	5.11 ± 1.01	0.621
MMSE score	21.07 ± 3.96	29.47 ± 1.29	<0.001
MoCA score	17.96 ± 3.57	26.83 ± 1.73	<0.001
Total Fazekas score	3.93 ± 1.14	0.14 ± 0.35	<0.001
PWMH	1.87 ± 0.76		
DWMH	2.07 ± 0.68		

*LVEF, left ventricular ejection fraction; MMSE, Mini-Mental State Examination; MoCA, Montreal Cognitive Assessment; PWMH, periventricular white matter hyperintensity; DWMH, deep white matter hyperintensity.*

### Comparison of DC Values Between CAD and HC

**FIGURE 1:**
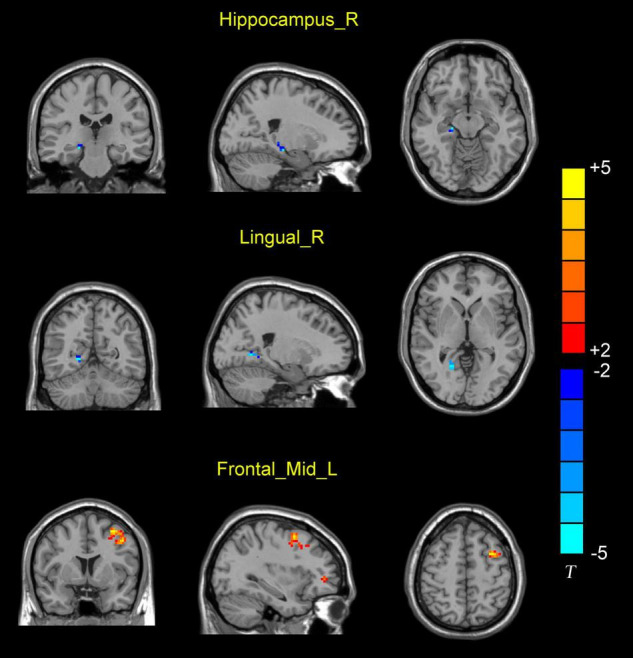
Differences in DC value between CAD patients and HC. Blue represents the decreased DC values in the right hippocampus (hippocampus_R) and right lingual gyrus (lingual_R). Red represents increased DC values in the left middle frontal gyrus (Frontal_Mid_L).

A total of 90 brain regions involved in Anatomical Automatic Labelling (ALL) were analyzed in the study. AAL partitions are provided by Montreal Neurological Institute (MNI). There are 116 regions in the AAL template but only 90 belong in the brain. Before comparison corrections, CAD patients showed significantly decreased DC value (*P* < 0.001; Table 2) in the right hippocampus (hippocampus_R), right lingual gyrus (lingual_R), left middle frontal gyrus (Frontal_Mid_L), left superior frontal gyrus (Frontal_Sup_L), and significantly increased DC values in the left middle frontal gyrus (Frontal_Mid_L) (*P* < 0.001). After comparison corrections, CAD patients showed significantly decreased DC values (*P* < 0.001; Table 2 and Figure 1) in the right hippocampus (hippocampus_R), right lingual gyrus (lingual_R), and significantly increased DC values (*P* < 0.001, Table 2 and Figure 1) in the left middle frontal gyrus (Frontal_Mid_L) when compared with healthy controls respectively.

**TABLE 2:** Brain regions with significantly decreased DC values in CAD patients compared with HC.

Brain regions	Voxels	BA	MNI coordinates			*P*-value
			
			X	Y	Z	
**Uncorrected**						
Frontal_Mid_L	10	47	–30	45	3	<0.001
Frontal_Sup_L	14	32	–18	36	27	<0.001
Hippocampus_R	15	36	21	–24	–12	<0.001
Lingual_R	15	30	21	–57	0	<0.001
Frontal_Mid_L	115	6	–33	6	54	<0.001
**Corrected**						
Hippocampus_R	15	36	21	–24	–12	<0.001
Lingual_R	15	30	21	–57	0	<0.001
Frontal_Mid_L	115	6	–33	6	54	<0.001

*Left middle frontal gyrus (frontal_mid_l), left superior frontal gyrus (frontal_sup_l), right hippocampus (hippocampus_R), right lingual gyrus (lingual_R), and left middle frontal gyrus (Frontal_Mid_L).*

### Correlation Between DC Values and Clinical Features in CAD Patients

Degree centrality value in the hippocampus_R significantly correlated (*P* < 0.001, Table 3) with MMSE and MoCA scores in CAD patients. Fazekas scores in CAD patients showed a significant correlation (*P* < 0.001, Table 3) with the DC value in the hippocampus_R.

**TABLE 3:** Correlation between DC values and clinical implications in CAD patients.

Variable	MMSE^a^		MoCA^a^		Fazekas score	
			
	β Coefficient (95% CI)	*P* value	β Coefficient (95% CI)	*P* value	β Coefficient (95% CI)	*P* value
Hippocampus_R	0.106 (0.047 –0.165)	<0.001	0.074 (0.044 –0.122)	<0.001	–0.598 (–0.821 –0.376)	<0.001
Lingual_R	–0.005 (–0.051 –0.041)	0.840	–0.036 (0.033 –0.091)	0.120	–0.116 (–0.304 –0.073)	0.230
Frontal_Mid_L	0.021 (–0.016 –0.057)	0.266	0.032 (0.022 –0.059)	0.167	0.118 (–0.035 –0.27)	0.131

*^a^Adjusted for age, hypertension, years of education, and gender.*

*Right hippocampus (hippocampus_R), right lingual gyrus (lingual_R), and left middle frontal gyrus (Frontal_Mid_L).*

## Discussion

Coronary artery disease is thought to affect the brain in multiple ways and recent reports have shown CAD may lead to cognitive impairment; it has been suggested cerebral hypoperfusion, and cardioembolism which may lead to cerebral atherosclerosis may be linked with the underlying mechanism (Xie et al., 2019). A current report suggested that there could be a gradual process at play affecting blood flow and the brain but how it works is still unclear (Rovio et al., 2019). To the best of our knowledge, this is the first study to assess the intrinsic dysconnectivity pattern of brain functional networks in CAD patients by using DC analysis. Our current report showed that CAD patients had significantly lower DC values in the right hippocampus, right lingual gyrus, and left middle frontal gyrus when compared with healthy controls. We also showed that reduced DC value in the right hippocampus correlated with reduced MoCA and MMSE scores and increased Fazekas score in CAD patients.

We observed a significant neural connectivity decrease in the right lingual gyrus of CAD patients compared with HCs. The lingual gyrus is a structure in the brain that is linked to visual processing and is part of the primary visual cortex of the brain (Zhang et al., 2016; Yu et al., 2018). It has been suggested that cardiovascular patients may be at a higher risk of developing visual-related problems (Flammer et al., 2013; Greenberg et al., 2015). Our report suggests that reduced neural connectivity in the right lingual gyrus may affect the vision of CAD patients which may explain the reduced visual acuity in CAD patients compared with healthy controls.

We also showed that CAD patients had significantly increased neural connectivity in the right middle frontal gyrus, which plays a significant role in the reorientation of attention in an individual (Japee et al., 2015). Therefore, we suggest that CAD may cause increased neural connectivity in the right middle frontal gyrus may be a compensatory effect by functional reorganization for the damaged brain tissue in this region to help with cognition.

The hippocampus has been well detailed to be involved in the commonest neurodegenerative disease, Alzheimer’s disease, leading to a significant decline in memory function (Allen et al., 2003; Jahn, 2013). This has been validated by implementing approaches that have shown decreases in glucose metabolism and perfusion in the hippocampus (Ishii et al., 2007; Mosconi, 2013). Significant changes in the hippocampus are evident not only in Alzheimer’s disease, characterized by impairment in everyday life but already in its pre-stage, mild cognitive impairment, where cognitive deficits are detectable in neuropsychological assessments without evident everyday life changes (Moon et al., 2018). Besides these functional changes, Schroeter et al. (2009) showed regional atrophy in the hippocampus of Alzheimer’s disease patients. Our current report showed CAD patients had significantly reduced neural connectivity in the right hippocampus when compared with healthy controls which may indicate functional dysfunction in the right hippocampus of CAD patients.

Taken together, both functional impairment and adaptation were observed in CAD patients. Decreased DC values (functional impairment) occurred in structures that play a role in visual memory (Lee et al., 2012; Zhang et al., 2016; Zammit et al., 2017); this may be due to cerebral hypoperfusion resulting from the decreased cardiac output as previously reported in CAD patients. Increased DC value was observed in the left middle frontal gyrus, suggesting functional plasticity to compensate for structural damage in the early phase of the disease (Mueller et al., 2020). These functional changes in CAD were associated with cognition, suggesting that neural connectivity changes may give clues to cognition dysfunction in the brain of CAD patients.

Neuropsychological assessments such as MMSE and MoCA have been shown to help assess the cognitive status of an individual. Our report showed a significant correlation between the reduced MMSE and MoCA scores and the reduced neural connectivity in the right hippocampus of CAD patients. Since the hippocampus plays a significant role in cognition (Rubin et al., 2014), the positive correlation between the reduced MoCA and MMSE scores and significantly reduced neural connectivity in the right hippocampus suggests that the reduced neural connectivity network in the right hippocampus reflects the cognitive assessment in CAD patients. Contrarily, visual stimulation is the main input for these neuropsychological assessments. The hippocampus is needed for the comprehension and execution of these neuropsychological examinations. Since the right hippocampus plays a significant role in comprehension, visual input, and visuospatial memories, reduced neural connectivity may affect the visual stimulation which may affect their cognitive status.

A previous report showed a significant correlation between non-calcified coronary plaque volume and total white matter hyperintensity volume (Kral et al., 2013); the authors suggested that coronary plaque in CAD patients affects the white matter lesions. Recently, it has been suggested that the association between cardiovascular diseases and white matter lesions may be linked to hypoperfusion in the cerebral microcirculation resulting from the reduced cardiac output, cardioembolism, and similar underlying risk factors (de Leeuw et al., 2000; Gurol, 2018). Our current report showed that reduced neural connectivity in the right hippocampus significantly correlated with increased white matter lesions on MR images using the Fazekas scale. White matter lesions suggest cerebral small vessel disease (which may be due to hypoperfusion) and are suggested to be linked with cognitive decline and small vessel disease (Wardlaw et al., 2015). Therefore, increased white matter lesions measured with the Fazekas scale and reduced neural connectivity in the right hippocampus may suggest that increased white matter lesions as a result of hypoperfusion may lead to reduced neural connectivity in the right hippocampus.

The pathological mechanism of cognitive decline in CAD patients is still unclear. In our current study, we utilized the functional magnetic resonance (DC sequence) to evaluate the neural connectivity in CAD patients, providing a possibility for further exploring the underlying mechanism. We would like to acknowledge some limitations in our work. To begin with, the cross-sectional design of our study and the small sample size of our participants limits us to conclude the cause and effect; longitudinal studies with larger sample sizes are needed to investigate more on our current study. As with most diagnostic tests, patient cooperation is an obligation. Movement from participants can diminish the quality of the image which may affect the data. Furthermore, we did not evaluate the microstructural integrity of the MRI images; further studies on the microstructural volume of participants may be needed. Our study focused on functional brain MRI measurements while cardiovascular measurement was not evaluated; further studies with a more comprehensive assessment of the heart may be needed. The clinical relevance of MRI procedures was investigated with cognitive parameters and white matter lesions; further studies are needed to assess the clinical importance of MRI measures and cardiovascular measures.

In conclusion, we showed that CAD patients have significantly reduced neural connectivity in the right hippocampus, right lingual gyrus, and left middle frontal gyrus when compared with healthy controls. We also showed that reduced DC value in the right hippocampus correlated with reduced MoCA and MMSE scores and increased Fazekas score in CAD patients. These findings suggest that reduced cerebral neural connectivity in CAD may contribute to their cognitive impairment and white matter microstructural damage.

## Data Availability Statement

The raw data supporting the conclusions of this article will be made available by the authors, without undue reservation.

## Ethics Statement

The studies involving human participants were reviewed and approved by First Affiliated Hospital of Zhejiang University School of Medicine. The patients/participants provided their written informed consent to participate in this study.

## Author Contributions

All authors listed have made a substantial, direct, and intellectual contribution to the work, and approved it for publication.

## Conflict of Interest

The authors declare that the research was conducted in the absence of any commercial or financial relationships that could be construed as a potential conflict of interest.

## Publisher’s Note

All claims expressed in this article are solely those of the authors and do not necessarily represent those of their affiliated organizations, or those of the publisher, the editors and the reviewers. Any product that may be evaluated in this article, or claim that may be made by its manufacturer, is not guaranteed or endorsed by the publisher.

## Footnotes

1
http://www.fil.ion.ucl.ac.uk/spm

2
http://restfmri.net/forum/

3
http://afni.nimh.nih.gov

## Supplementary Material

Supplementary Figure 1 Flow chart of the inclusion of participants for the study.
