# Supplementary figures and images for: Neural Dysconnectivity in the Hippocampus Correlates With White Matter Lesions and Cognitive Measures in Patients With Coronary Artery Disease
Source: Front Aging Neurosci. 2022 Jun 27;14:786253. doi: 10.3389/fnagi.2022.786253 (PMC9271740; doi:10.3389/fnagi.2022.786253)

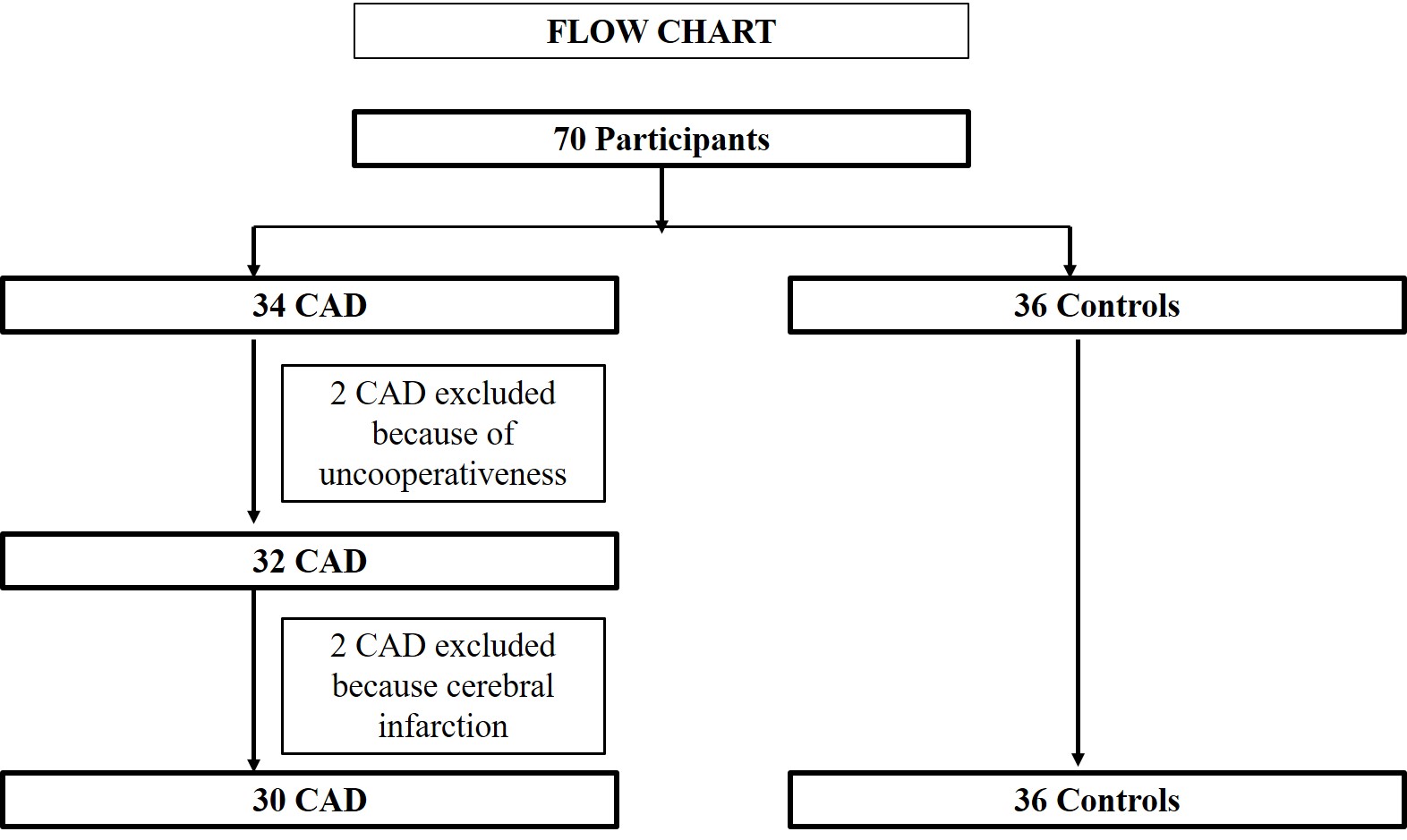

Supplement: Supplementary Figure 1 — Flow chart of the inclusion of participants for the study. [file Image_1.jpg]
